# Gene Flow within and between Catchments in the Threatened Riparian Plant *Myricaria germanica*

**DOI:** 10.1371/journal.pone.0099400

**Published:** 2014-06-16

**Authors:** Silke Werth, Christoph Scheidegger

**Affiliations:** 1 Swiss Federal Research Institute WSL, Birmensdorf, Switzerland; 2 University of Iceland, Dept. Life- and Environmental Sciences, Reykjavik, Iceland; CNR, Italy

## Abstract

One of the major distinctions of riparian habitats is their linearity. In linear habitats, gene flow is predicted to follow a one-dimensional stepping stone model, characterized by bidirectional gene flow between neighboring populations. Here, we studied the genetic structure of *Myricaria germanica*, a threatened riparian shrub which is capable of both wind and water dispersal. Our data led us to reject the ‘one catchment – one gene pool’ hypothesis as we found support for two gene pools, rather than four as expected in a study area including four catchments. This result also implies that in the history of the studied populations, dispersal across catchments has occurred. Two contemporary catchment-crossing migration events were detected, albeit between spatially proximate catchments. Allelic richness and inbreeding coefficients differed substantially between gene pools. There was significant isolation by distance, and our data confirmed the one-dimensional stepping-stone model of gene flow. Contemporary migration was bidirectional within the studied catchments, implying that dispersal vectors other than water are important for *M. germanica.*

## Introduction

Riparian habitats host a rich assemblage of specialist plant species confined to floodplains [1]. Due to the degradation, loss and fragmentation of their natural habitats, many of the species specialized on riparian habitats have declined severely during the last centuries, leading to drastic reductions in population size or to local extinctions [2]–[4].

One of the important characteristics distinguishing riparian from other habitats is their linearity. Linear habitats may function as corridors, facilitating rapid movement of individuals and genes across a landscape [5]. Gene flow is an important process in riparian plant populations because the movement of genes through propagules and gametes ensures connectivity of upstream and downstream populations [6], [7]. In plants, gene flow is mediated by seeds, vegetative propagules such as shoots, as well as pollen [8]. Hydrochory, the dispersal of propagules with the water flow of a river, is an important process promoting species richness of riparian habitats [1], [9], [10]. Water-dispersed propagules are exclusively distributed downstream, and within a given catchment. Alternatively, transport of propagules is possible via animal vectors (zoochory) or wind (anemochory) [11]; these vectors can transport propagules upstream and downstream. In insect and wind pollinated species, gene flow via pollen can occur both in upstream and in downstream direction along a river, and across catchments.

In plants, quantifying migration is a notoriously difficult task, because it is often not possible to observe the dispersal of propagules directly [8], [12], [13]. However, contemporary migration can be assessed using assignment tests that rely on population genetic data [14], [15]. These tests identify which individuals are migrants, and from which population they derive or, alternatively, if they originate from outside of the sampled populations. Knowing the source population of a migrant allows assessing the directionality of gene flow – e.g. if it is mainly directed downstream or if there is some upstream migration.

The various ways for migration to occur in plant populations allow us to test an explicit hypothesis on gene flow. Our first hypothesis states that gene flow is mainly directed downstream, as expected if hydrochory is the most important dispersal mode. Our alternative hypothesis is that gene flow should be bidirectional as predicted in a riparian species dispersed mainly by a combination of vectors including water, wind, and animals. We approached testing the null hypothesis by making use of migrate-n, a powerful software that allows the quantification of bidirectional migration rates and population sizes in a coalescent framework using Markov Chain Monte Carlo computing, and by quantifying contemporary migration with assignment tests using the software GeneClass2, which can be used to identify and assign first-generation migrants to source populations. In migrate-n, apart from testing upstream vs. downstream stepping-stone migration models, we tested whether populations in a catchment were consistent with a single panmictic population. Last but not least, we tested whether there was statistical support for an island model (gene flow bidirectional and occurring between all populations).

Our second hypothesis relates to the spatial distribution of gene pools in multiple catchments. We hypothesize that in a plant species dispersed via water, there should be genetic divergence between populations from different catchments because the crossing of catchments would not be feasible with this dispersal vector. Hence, if the populations of a riparian plant in multiple catchments have remained isolated over many generations, each catchment should be populated by its unique gene pool (‘one catchment – one gene pool’ hypothesis). Alternatively, gene flow by other vectors than water would lead to the spatial distribution of gene pools across multiple catchments.

Our third objective was to explore patterns of genetic diversity across space and between genetic clusters. We hypothesized that genetic diversity should be related to elevation, with highest diversity in downstream sites as a consequence of seed dispersal with water. Moreover, in agreement with population genetic theory, larger populations should harbor more genetic diversity than smaller populations and downstream populations could be larger owing to the immigration of individuals from upstream sites. Finally, we analyzed the mating system using population-specific inbreeding coefficients [16]. Mating system is an important factor influencing population subdivision and genetic diversity of plant populations [8]. The inbreeding coefficient of an individual relative to that of its subpopulation (*F*_IS_) provides important insight into the mating system. Genetic diversity of populations may be influenced by mating system. If local population size has remained small over several generations, selfing leads to an increased frequency of homozygous individuals and may lead to a loss of rare alleles over time due to random sampling effects. Here, we tested specifically whether there was *i*) a relationship between *F_IS_* and affiliation to gene pool, and *ii*) whether high-elevation sites exhibited a different level of inbreeding than low-elevation sites.

Our fourth hypothesis concerned isolation by distance [17]. Stream habitats are linear environments and the movement of propagules should occur in a linear fashion, i.e. along one dimension in space [18]. A one-dimensional stepping-stone model does accurately describe gene flow in such systems. The stepping-stone model is characterized by gene flow upstream and downstream between neighboring populations. Based on simulations, Slatkin demonstrated that the slope of the regression of log_10_ of 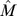 (gene flow) over log_10_ of geographic distance should be about −1.0, if gene flow occurs mainly in one dimension in space, as expected in linear habitats [19]. In contrast, in the case of two-dimensional gene flow, the slope of the regression is expected to be circa −0.3. The intercept of the regression can be utilized to estimate the effective number of migrants Nm, which can be interpreted as neighborhood size [19].

*Myricaria germanica* (Tamaricaceae) is a threatened riparian shrub growing on gravel banks along rivers. In Central Europe, this character plant of riparian vegetation [20], [21] has declined severely owing to habitat loss associated with river channelization and gravel extraction during the past century [22]. The species requires habitats which are flooded not more frequently than every seven years [23], and it is a habitat specialist requiring dynamic, braided rivers. Details of the mating system in this insect-pollinated, hermaphroditic plant are not known, but another species of the genus *Myricaria* is able to self [24].

Here, we used population genetic analyses of a large dataset of microsatellite genotypes to understand regional patterns of gene flow and genetic diversity in *M. germanica*, and to determine which model of migration fits this species best in catchments of major rivers in Switzerland.

## Results

### Directionality of gene flow

Analysis of contemporary migration based on first-generation migrants using the software GeneClass2 revealed a number of first-generation migrants within the Inn and Rhine catchments, with migration being directed both upstream and downstream (Table 1). The number of migration events did not differ significantly between upstream and downstream direction, as assessed with a one-sided, paired Student's *t*-test assuming unequal variance among groups (*t* = 0.2255, df = 3, *p* = 0.42; mean upstream: 5.25; mean downstream: 5.75).

**Table 1:** Analysis of contemporary migration within and among catchments (software GeneClass2).

Migration between sampled sites		
Nr. migrants	Direction	Catchment
12	Downstream	Inn
7	Upstream	Inn
5	Downstream	Rhine
10	Upstream	Rhine
1	Downstream	Rhone
2	Upstream	Rhone
5	Downstream	Ticino*
2	Upstream	Ticino*

Migration from outside the sampled sites		
Nr. migrants	Direction	Catchment
6	Unknown	Inn
13	Unknown	Rhine
6	Unknown	Rhone
3	Unknown	Ticino

*In addition, one event between catchments (from Inn to Ticino).

The table gives the number of migrants and the direction of migration in each catchment, assessing migration between the sampling sites and listing migrants that had a high likelihood to originate from outside of the sampled sites.

Model selection based on natural logarithmic Bayes Factors in analysis of recent migration with Migrate-n gave support for migration being directed downstream in a stepping-stone fashion in the Rhine catchment (Table 2). For the Maggia and Rhone catchments, downstream models of gene flow did not converge, even if they were run tenfold longer (data not shown), indicating poor model fit. Hence, downstream models were not considered for calculating Bayes Factors. For the the Inn, Maggia, and Rhone catchments, panmixia was inferred based on Bayes factors.

**Table 2:** Log Bayes factors and model parameters from analysis of migration (software Migrate-n) in the riparian shrub *Myricaria germanica* collected from four catchments in Switzerland.

Bayes Factors (LBF)					
Catchment	Full	Step bidir	Downstream	Step downst	Panmixia
Inn	−5,788,873	−3,493,117	−688,498	−786,023	0***
Maggia	−314,121	—	NC	—	0***
Rhine	−6,003,175	−735,991	−11,927	0***	−182,051
Rhone	−412,522	—	NC	—	0***

Model parameters					
Catchment	Migration rate		Population size		
Inn	—		1625.3		
Maggia	—		566.9		
Rhine	M_1→2_	8.2	Pop1 550.2		
	M_2→3_	36.1	Pop2 150.3		
	M_3→4_	30.4	Pop3 416.9		
			Pop4 250.3		
Rhone	—		991.9		

Bayes Factors were constructed in comparison with the model with the largest log likelihood (Bayes Factor zero). Model probability was calculated by dividing the marginal likelihood of a given model by the sum of the marginal likelihoods of all models. Model probabilities:

*0.01<*s*_i_<0.05;

**0.05<*s*_i_<0.10;

***0.95<*s*_i_<1.00.

NC, no convergence of model, thus excluded for calculation of Bayes Factors. Migration rate estimates and population sizes of transformed data are shown in the lower panel. Populations were sorted in downstream order with increasing number, i.e. Pop1 was the most upstream. M_1→2_ denotes the migration rate from population 1 to population 2.

### Population subdivision and spatial distribution of gene pools

**Figure 1:**
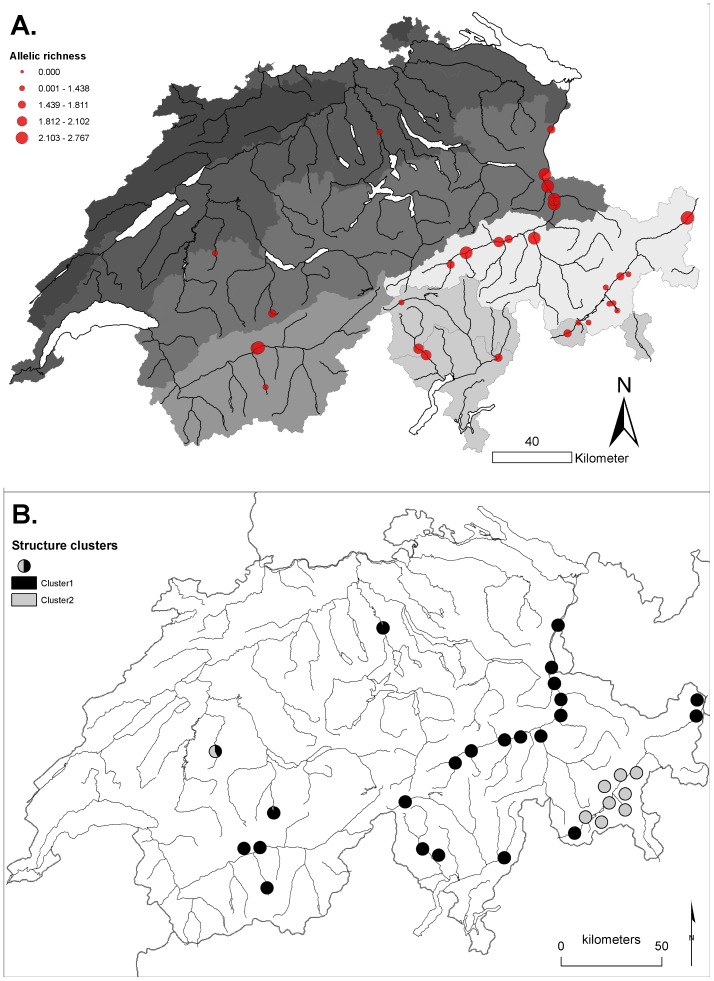
Allelic richness and cluster affiliations of the studied sites of *Myricaria germanica* (Tamaricaceae) in Switzerland. A. Allelic richness. The shading of the map shows biogeographic regions of Switzerland, as used in analysis of molecular variance. B. Results from Bayesian analysis of population structure. Map data: modified from Vector25 © 2011 swisstopo (contract number 5704000000); biogeographic regions: modified following data from BAFU, CH-3003 Bern, Switzerland.

Our data exhibited a high amount of genetic differentiation between populations. Analysis of molecular variance revealed significant genetic structure due to the grouping of sites by river (31.9% of total variance, Table 3). There was also significant variance due to populations within groups, and individuals within populations. Bayesian analysis of population structure revealed two distinct gene pools (‘clusters’) in the four catchments of our study area (Fig. 1B). Cluster 1 was frequent in Rhine, Rhone, and Ticino catchments, but rare in the Inn catchment. Cluster 2 occurred mainly in the Inn catchment, with single occurrences in the Ticino (MÄI1) and Rhine (SEN1) catchments.

**Table 3:** Analysis of molecular variance in populations of the threatened riparian shrub *Myricaria germanica*, grouping sites by river.

Source	Df	SS	Varcomp	Perc
**Grouping by river**				
Between rivers	11	5484.4	2.041	31.9
Between sites within rivers	19	2668.8	1.947	30.4
Between individuals within sites	1083	3605.5	0.918	14.4
Between individuals	1114	1663.5	1.493	23.3
Total	2227	13422.2	6.399	100.0

*F*-statistic	Value			
*F*_CT_	0.319*			
*F*_SC_	0.447*			
*F*_IS_	0.381*			
*F*_IT_	0.767*			

The table gives the source of variability, the degrees of freedom, the sum of squares, the variance component, the percentage of variation, and the value of the *F*-statistic.

*, p<0.001.

**Figure 2:**
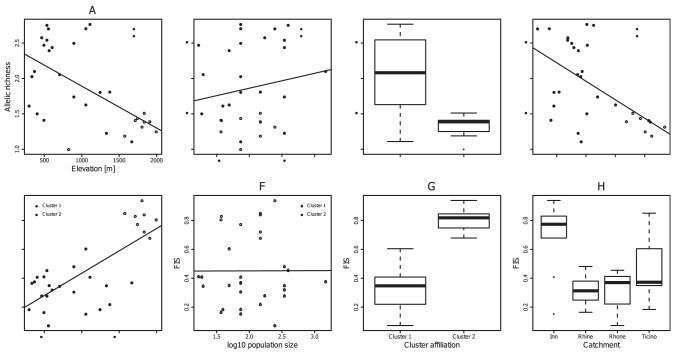
Population graphs showing the genetic relationships between *Myricaria germanica* sites, with catchments shown in different colors (Inn: blue; Rhine, orange; Rhone, red; Ticino, yellow); the size of circles is proportional to effective population size.

The population graph approach revealed two disconnected subnetworks which represented the same groups of individuals detected with Bayesian analysis of population structure (Fig. 2), thus refuting the ‘one catchment, one gene pool’ hypothesis. The first subnetwork comprised seven sites within the Inn catchment and one spatially proximate site (MÄI1) situated in the Ticino catchment. However, two of the sites belonging to the Inn catchment at the border to Austria belonged to the second subnetwork, same as in Bayesian analysis of population structure. Sites situated within the Rhine catchment were well connected to other sites, whereas most sites from the Rhone and Ticino catchments exhibited rather few links to other sites (Fig. 2).

In agreement with the results from Bayesian analysis of population structure and population graphs, analysis of pairwise *F*_ST_ values showed that the average *F*_ST_ was higher between (average = 0.72; minimum = 0.55, maximum = 0.97) than within clusters (Cluster1, average = 0.51; Cluster 2, average = 0.24). Within Cluster 1, *F*_ST_ values ranged from 0.01 to 0.98; within Cluster 2, they ranged from 0 to 0.44.

### Geographic patterns of genetic diversity and inbreeding

Properties of the collecting sites including their allelic richness and inbreeding coefficients are given in Table 4. In the 31 populations analyzed across an elevation gradient of 1660 m, Nei's gene diversity ranged from 0 to 0.427, and mean allelic richness from 1 to 2.767.

**Table 4:** Summary statistics of the sites included in the analysis of *Myricaria germanica*.

Pop	Biogeographic region	Catchment	River	N	X	Y	Elev	Pop	H_E_	A_R_	*F*_IS_
BED1	South side of Alps	Ticino	Ticino	35	8.524550	46.505080	1335	350	0.090	1.229	0.350
INN1	Eastern Central Alps	Inn	Ova da Bernina	39	9.919276	46.480341	1840	150	0.137	1.388	0.841
INN2	Eastern Central Alps	Inn	Beverin	40	9.871962	46.552636	1750	75	0.160	1.439	0.774
INN3	Eastern Central Alps	Inn	Ova da Roseg	40	9.892024	46.478693	1810	250	0.123	1.316	0.939
INN4	Eastern Central Alps	Inn	Ova da Morteratsch	40	9.942740	46.446552	1915	150	0.113	1.393	0.678
INN5	Eastern Central Alps	Inn	Inn	40	9.970937	46.601308	1840	150	0.153	1.512	0.722
INN6	Eastern Central Alps	Inn	Inn	39	10.430319	46.858684	1060	75	0.413	2.707	0.153
INN7	South side of Alps	Inn	Ova da Fedox	9	9.751708	46.396107	2000	37.5	0.086	1.250	0.806
INN8	Eastern Central Alps	Inn	Inn	34	10.424082	46.853896	1115	75	0.425	2.767	0.408
INN9	Eastern Central Alps	Inn	Ova da Varusch	38	10.023618	46.610131	1720	38	0.132	1.410	0.830
KAN1	North side of Alps	Rhine	Kander	37	7.674966	46.460681	1380	150	0.230	1.812	0.219
MAG1	South side of Alps	Ticino	Maggia	38	8.682544	46.265588	340	75	0.339	2.029	0.367
MAG2	South side of Alps	Ticino	Maggia	39	8.632446	46.294008	374	1500	0.352	2.103	0.377
MÄI1	South side of Alps	Ticino	Maira	43	9.684693	46.397940	1580	150	0.062	1.189	0.851
MÄI2	South side of Alps	Ticino	Maira	56	9.612635	46.349940	1060	50	0.140	1.630	0.605
MOE1	South side of Alps	Ticino	Moesa	36	9.156040	46.247523	302	40	0.221	1.615	0.184
MRH1	Eastern Central Alps	Rhine	Mittelrhein	40	8.854504	46.674300	1250	75	0.280	1.808	0.185
REU1	North side of Alps	Rhine	Reuss	19	8.394524	47.281451	500	37.5	0.154	1.413	**0.164**
RHE1	North side of Alps	Rhine	Alpenrhein	33	9.546762	46.939694	540	350	0.384	2.545	0.278
RHE2	Eastern Central Alps	Rhine	Hinterrhein	40	9.410571	46.786434	610	350	0.338	2.440	0.320
RHE3	North side of Alps	Rhine	Alpenrhein	44	9.491499	47.073971	470	175	0.390	2.579	0.279
RHE4	North side of Alps	Rhine	Alpenrhein	40	9.547920	46.960477	570	50	0.358	2.397	0.351
RHE5	North side of Alps	Rhine	Alpenrhein	18	9.541920	47.277910	410	19	0.075	1.504	0.409
RHE6	North side of Alps	Rhine	Alpenrhein	17	9.507980	47.019300	500	17	0.395	2.473	0.412
RHO1	Western Central Alps	Rhone	Rhone	44	7.575609	46.302413	540	400	0.427	2.755	0.455
RHO2	Western Central Alps	Rhone	La Navisence	38	7.630048	46.126273	1674	75	0.029	1.109	0.370
RHO3	Western Central Alps	Rhone	Rhone	36	7.585912	46.306314	560	250	0.392	2.704	0.073
SEN1	North side of Alps	Rhine	Sense	39	7.296919	46.734403	829	75	0.000	1.000	—*
VRH1	Eastern Central Alps	Rhine	Vorderrhein	41	9.240862	46.785215	900	350	0.259	1.743	0.482
VRH2	Eastern Central Alps	Rhine	Vorderrhein	21	9.175004	46.772721	710	20	0.302	2.059	0.346
VRH4	Eastern Central Alps	Rhine	Vorderrhein	41	8.957355	46.727076	900	75	0.422	2.500	0.306

*Population monomorphic.

The table gives the population name, the number of samples analyzed, the GPS coordinates (map datum WGS84), elevation [m], the midpoint of the estimated interval of population size (Pop), gene diversity H_E_, allelic richness A_R_, and the inbreeding coefficient *F*_IS_ (non-significant value in bold).

**Figure 3:**
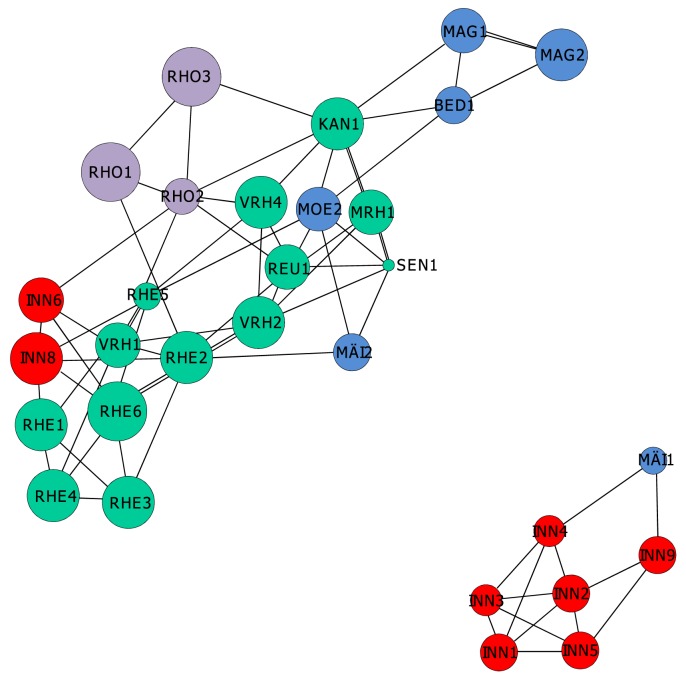
Allelic richness and population-specific inbreeding coefficients (*F*_IS_) of 20 nuclear SSR in *Myricaria germanica*. **A and E.** Relationship with elevation. B and F. Relationship with log10-transformed census population size. D. Relationship between allelic richness and *F*_IS_. C and G. Boxplots of allelic richness, grouped by affiliation to genetic clusters (see Fig. 1B). H. Boxplots of FIS, grouped by affiliation to catchment. The values plotted for census population size are midpoints of the estimated intervals of population size. Lines represent linear regressions. Cluster 1: Rhine, Rhone, Ticino. Cluster 2: Inn.

A clear geographic trend in allelic richness was visible in our data: Populations located in the Engadine, the valley of the river Inn in southeastern Switzerland had lower allelic richness than populations from other regions (Fig. 3). These sites formed a separate gene pool (Cluster 2), as determined from Bayesian analysis of population structure.

The best linear model as determined by AIC predicting allelic richness included the sites' affiliation to genetic clusters identified by Bayesian analysis of population structure (Table 5). We found a significant relationship between elevation and allelic richness: high-altitude populations of *M. germanica* had lower genetic diversity than low or middle altitude populations (Fig. 3A, Table 3). However, this relationship was most likely the effect of confounding, i.e. the low-diversity Cluster 2 populations occurring at high elevations, which showed a higher level of inbreeding (Fig. 3C, Fig. 3G). As expected in a selfing plant, allelic richness decreased with increasing levels of inbreeding (Fig. 3D). There was no significant relationship between allelic richness and log-transformed population size (Fig. 3B).

**Table 5:** Linear regression models of allelic richness A_R_ (response), inbreeding coefficient *F*_IS_ (response), cluster affiliation, elevation, and log_10_-transformed population size (log10_._Pop.size).

Model	Parameters	df	SS	MS	*F*	p-value	AIC
**Model : A_R_ ∼ Cluster**							
	Cluster	1	3.742	3.742	17.9	0.00021	43.3
	Residuals	29	6.052	0.209			
**Model : A_R_ ∼ Elevation**							
	Elevation	1	3.361	3.361	15.2	0.00054	45.2
	Residuals	29	6.433	0.222			
**Model: A_R_ ∼ log10.Pop.size**							
	Pop.size	1	0.269	0.268	0.8	0.37330	57.4
	Residuals	29	9.525	0.328			
**Model : A_R_ ∼ Cluster + Elevation**							
	Cluster	1	3.742	3.742	18.3	0.00020	43.6
	Elevation	1	0.330	0.330	1.6	0.21410	
	Residuals	28	5.722	0.204			
**Model : A_R_ ∼ Elevation + Cluster**							
	Elevation	1	3.361	3.361	16.4	0.00036	43.6
	Cluster	1	0.711	0.711	3.5	0.07261	
	Residuals	28	5.722	0.204			
**Model: A_R_ ∼ log10.Pop.size + ** ***F*** **_IS_**							
	log10.Pop.size	1	0.220	0.220	1.0	0.32200	44.0
	*F*_IS_	1	2.971	2.971	13.8	0.00095	
	Residuals	27	5.830	0.216			
**Model: ** ***F*** **_IS_ ∼ Cluster**							
	Cluster	1	1.369	1.369	103.7	6.44E-11	−40.8
	Residuals	28	0.370	0.013			
**Model: ** ***F*** **_IS_ ∼ Elevation**							
	Elevation	1	0.914	0.914	31.0	0.00584	−16.7
	Residuals	28	0.824	0.029			
**Model: ** ***F*** **_IS_ ∼ log10.Pop.size**							
	Pop.size	1	0.000	0.000	0.000	0.98740	5.7
	Residuals	28	1.738	0.062			
**Model: ** ***F*** **_IS_ ∼ Elevation + Cluster**							
	Elevation	1	0.914	0.914	66.8	8,85E-09	−38.8
	Cluster	1	0.455	0.455	33.3	3,89E-06	
	Residuals	27	0.369	0.014			
**Model: ** ***F*** **_IS_ ∼ Cluster + Elevation**							
	Cluster	1	1.369	1.369	100.1	1,41E-10	−38.8
	Elevation	1	0.000	0.000	0.0	0.87200	
	Residuals	27	0.369	0.014			

The table gives the degrees of freedom (df), the sum of squares (SS), mean square (MS), the *F*-value (*F*), the significance of the respective parameter (*p*-value) and Akaike's information criterion (AIC).

The values of the inbreeding coefficient *F*_IS_ ranged from low to high (0.072 to 0.939), indicating variation in mating system across sites (Table 4) and catchments (Fig. 3H). Sites belonging to Cluster 1 were consistent with a mixed (*F*_IS_ = 0.15 to 0.48) or outcrossing mating system (*F*_IS_ = 0.07). Sites belonging to Cluster 2 exhibited high inbreeding coefficients (*F*_IS_ = 0.68 to 0.94, Table 4; Fig. 3G). There was no relationship between *F*_IS_ and census population size (Fig. 3F). We found a significant positive relationship of the population-specific inbreeding coefficient, *F*_IS_, with elevation, indicating that *F*_IS_ and allelic richness covaried along the elevational gradient, but allelic richness decreased with elevation.

### Isolation by distance

**Figure 4:**
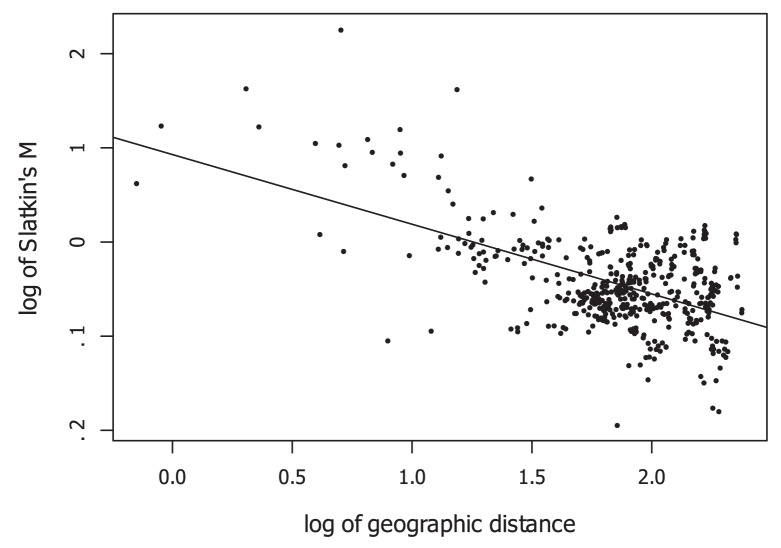
Log10 (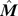) plotted against log10(geographic distance in km) for the threatened riparian shrub *Myricaria germanica* based on samples from 31 sites in Switzerland. The linear regression equation used to plot the line was log10(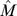) = 0.93−0.74×log10(geographic distance), with an adjusted *R* ^2^ of 0.314.

The Mantel test indicated that there was a significant relation between genetic, i.e. *F*_ST_/(1-*F*_ST_) and geographic distance (*r*_M_ = 0.22, p = 0.001). Gene flow as estimated by the log_10_ of 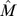 decreased with the log_10_ of geographic distance d according to the equation log_10_(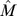) = 0.93−0.74*log_10_(d) (Fig. 4). The slope of the relationship was consistent with the expectation under a one-dimensional stepping stone model (range of expected values: −0.5 to −1.5 versus −0.5 to −0.15 in a two-dimensional model [19]). Based on the intercept of the regression line, Nm (neighborhood size) was estimated to be 8.5 for our study species [19].

## Discussion

Our data showed that contemporary gene flow in *M. germanica* was bidirectional, whereas historic gene flow was directed downstream in the Rhine catchment. Our data rejected the ‘one catchment-one gene pool’ hypothesis, as a single genetic cluster was distributed across four catchments and another one across two. Population graph analysis showed that there were no connections between the two clusters found, highlighting their genetic isolation. Sites situated in the southeast of Switzerland were characterized by low diversity, high inbreeding coefficients, and were differentiated from all remaining sites, belonging to a separate genetic cluster. Last but not least, our data showed significant isolation by distance, supporting a one-dimensional stepping-stone model.

### Directionality of contemporary and historic gene flow

Contemporary gene flow, as estimated from analysis of first generation migrants based on assignment tests, took place mainly within catchments, with two exceptions where gene movement was detected between catchments in sites that were spatially proximate (from the Engadine to Bergell valley). Hence, dispersal between catchments is possible in *M. germanica*. Few other studies have found evidence for contemporary dispersal between catchments in riparian plant populations.

Moreover, contemporary gene flow occurred both in upstream and downstream direction in *M. germanica.* Also isolation by distance analysis supported bidirectional migration (see below). While migration events directed downstream are most likely to have arisen from hydrochory of seeds in combination with wind dispersal, upstream migration must have taken place either by wind or animals. It is unlikely that long-distance pollen dispersal events would have been reported as migrants, as the method only allowed detecting individuals with both gene copies in the source population.

Several studies have found support for either bidirectional gene flow or a source/sink scenario [7], [25], [26]. In the riparian shrub *Myricaria laxiflora*, unidirectional gene flow downstream and considerable genetic differentiation between populations has been reported [24], suggesting that gene flow followed a source-sink model. In the aquatic macrophyte *Sparganium emersum*, an accumulation of genetic diversity in downstream populations was found, together with high differentiation between populations pointing towards source-sink population dynamics [27]. One study investigated the directionality of gene flow in three plant species along one river, and performed a meta-analysis of published studies [28]. No evidence for unidirectional gene flow was found, neither in any of the study species, nor in the metaanalysis. Our data show a different pattern than those of *M. laxiflora*, which showed evidence for linear, unidirectional migration via hydrochory [24].

Contemporary migration was bidirectional. In contrast,historic gene flow was directed downstream in the largest catchment, Rhine. Contemporary and historic directionalities of gene flow may differ for several reasons. Historic gene flow reflects the main directionality of gene flow over a long time, and support for the directionality downstream does not mean that there have never been any events in the other direction. Individuals dispersed by the vectors wind/animals could have lower reproductive success in the populations they are dispersed to, and then their genes may not be traced in historic signal. Moreover, we can not rule out that the importance of individual dispersal vectors may have changed over time. For example, it could well be that some dispersal events represent recent human-aided dispersal in the framework of conservation translocations, which would lead to a discrepancy among contemporary and historic directionalities.

Along three catchments, model selection in Migrate-n based on Bayes Factors provided evidence of panmixia. For the Inn catchment, this result seems plausible as genetic differentiation between sites was generally low. For Rhone and Maggia, however, the result of panmixia is in conflict with the strong population subdivision evident from *F*_ST_ values and AMOVA that is typical for selfing plant populations. Since the downstream models of gene flow did not converge in these two cases, we can not fully exclude the possibility that model selection in Migrate-n based on Bayes Factors inferred an erroneous model.

Our historic gene flow analysis highlights the importance of water and wind in seed dispersal for the Rhine catchment. Several other studies have emphasized the importance of seed dispersal via hydrochory in riparian and aquatic plants [1], [10], [27], [29]–[34]. Wind may occasionally transport seeds over large distances, but long-distance dispersal events are not frequent [35]–[37]. A study of three riparian plants showed that in none of the species, gene flow was unidirectional [28]. In the sites we studied in Switzerland, contemporarily, wind or animal-mediated dispersal appears to be equally important as hydrochory.

### Population subdivision and spatial distribution of gene pools

It is obvious from Bayesian analysis of population structure, analysis of molecular variance, population graphs, and from the contemporary pattern of migration that the sites sampled for *M. germanica* do not form a single continuous population. We found substantial population subdivision between and within rivers; this result is similar to what was found in other studies of riparian and aquatic shrubs or herbs [7], [24], [27], [38]–[40]. Our study species exhibited far more population subdivision than two wind-pollinated riparian tree species [34], [41]. We attribute this difference partly to efficient pollen dispersal by wind in these trees, increasing gene flow between populations. Moreover, trees have a larger release height of seeds than shrubs such as *M. germanica*, hence their capability for long-distance seed dispersal by wind should be greater [42]–[44]. The strong population structure in *M. germanica* can likely be explained by frequent selfing. In selfing plant species, pollen dispersal is low, leading to population structure unless seed dispersal is highly efficient. Moreover, selfing reduces effective population size, thus increasing drift and leading to higher degrees of population subdivision [45].

Our data rejected the one-catchment, one gene pool hypothesis, under which we would have expected four genetic clusters to occur, each in one catchment. Instead, there were only two clusters, and the same cluster occurred in multiple catchments. None of the studies we examined for riparian and aquatic plant populations found support for the one-catchment, one gene pool hypothesis. Several studies reported multiple gene pools of riparian and aquatic plants in a single catchment [7], [24], [27], [34]. A few studies have analyzed the spatial distribution of gene pools of riparian plants in multiple catchments. In two studies, gene pools of riparian plants were distributed across multiple catchments [39], [40]. One of these studies reported that the spatial distribution of two gene pools of the riparian shrub *Rhododendron ripense* corresponded to Pleistocene river systems [39], thus highlighting the importance of population history.

The spatial distribution of a single gene pool across multiple catchments in *M. germanica* implies that there must have been catchment-crossing dispersal events at some time in the history of Cluster 1, founding populations in different catchments. Moreover, there must have been at least one historic dispersal event among catchments for Cluster 2 which also spans across two catchments in southeastern Switzerland.

### Geographic patterns of genetic diversity

The arguably most striking pattern with respect to genetic diversity found in the data was the vast discrepancy in genetic diversity between Clusters 1 and 2. Cluster 2 sites located in the Engadine valley in southeastern Switzerland exhibited a far lower diversity than all remaining sites, with the notable exception of two (SEN1, RHO2).

One result of interest is the vast discrepancy of inbreeding coefficients across sites, indicating geographic variation in mating system. A high level of inbreeding was inferred for sites belonging to Cluster 2 (Engadine). The only other species of *Myricaria* investigated with population genetic approaches to date, *M. laxiflora*, was determined to be predominantly selfing in a study that used amplified fragment-length polymorphisms (AFLPs) to investigate genetic variability in populations of the Yangtze River in China [24]. Based on our data, we conclude that *M. germanica* has a mixed mating system, with frequent selfing. When doing hand-pollinations to make crosses of plants from different catchments (Inn vs. Rhine), a number of the progeny turned out to be selfed, rather than out-crossed (Werth & Scheidegger, unpublished data).

Contrary to the theoretical expectation [46], we found no evidence for a relationship between population census size and genetic diversity. Several other studies of riparian plants have found the same pattern for riparian plants [7], [24] and for riparian populations of a grassland plant [47]; some of these authors have interpreted this result as evidence for lack of regional equilibrium, as expected in a metapopulation. The genetic diversity of sites may reflect the mating system of a plant population: Low genetic diversity is expected for frequently inbreeding populations [48] such as those of selfing plants. Indeed, confirming this expectation, we found a significant negative relationship between allelic richness and *F*_IS_. Low genetic diversity could also result from recent changes in population size, e.g. bottlenecks or founder events after the colonization of new habitat patches [7], [49].

We found significant relationships with elevation in allelic richness and *F*_IS_. Our linear models indicated that this effect is likely due to the confounding effect of the highly inbred, low-diversity Engadine sites being located at high elevations.

### Isolation by distance

We found statistical support for isolation by distance in the studied sites; genetic differentiation (pairwise standardized *F*_ST_) followed a linear relationship with geographic distance as determined by a Mantel test. Most of the prior studies of riparian and aquatic plants did not find isolation by distance [7], [27], [33], [34], [50], a meta-analysis is presented in [28]. Only few studies found a significant relationship between genetic and geographic distance in riparian and aquatic plants [6], [24], [39]. For the riparian herb *Ainsliaea faurieana*, isolation by distance was only found when several catchments were analyzed in combination, but not within a single river [40]. In other studies, isolation by distance was found in only one of three studied species [28], or in one of three catchments [18].

Based on the regression of log_10_(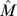) on log_10_ of geographic distance, our data are consistent with a one-dimensional stepping-stone model, in which gene flow is bidirectional and occurs only among neighboring populations [19]. The directionality of migration is consistent with the bidirectional contemporary migration revealed by assignment tests. However, the assignment tests revealed migration events that extended beyond neighboring populations.

## Materials and Methods

### Study species

*Myricaria germanica* is a riparian shrub which occurs along natural and near-natural rivers in Europe and Asia. Maximum ages of 21 years [51] and 70 years [52] have been reported. The species' natural world-wide distribution is restricted to mountainous regions of Europe and Asia, i.e. the Alps, Pyrenees, Scandes, Apennine, Carpathians, Caucasus, and Himalaya [53]. The Himalaya region is the centre of origin of the genus *Myricaria*, and harbours multiple species of the genus [54]. As the only species of its family naturally occurring in Switzerland (*Tamarix* spp. are sometimes used as ornamental plants), *M. germanica* grows on gravel banks along rivers from the colline to the subalpine altitudinal zone (500–2100 m). Being a pioneer species on gravel bars, *M. germanica* forms patchy populations, with frequent colonizations of new patches and extinction of existing populations after disturbance by flooding. When rivers are channelized in a way that suitable habitat, in particular sites with intermediate disturbance frequencies are lacking, the species can go locally extinct. Thus, *M. germanica* has faced a severe decline in many of the major rivers of Europe in the past decades. Once a rather common species on the Swiss Plateau, the species is now restricted to a few sites in this region. In Switzerland, *M. germanica* is most common in the Southeast, i.e. the Cantons of Grison and Ticino (Fig. 1).

### Study area, sampling design, and molecular analysis

Our sampling included 1114 samples collected from 31 sites situated in all geographic regions where *M. germanica* is known to occur in Switzerland (Table 1, Fig. 1). The local density of sampling sites reflects the number of populations of the species in a particular catchment. Tissue samples were collected from the apical tips of branches without flowers, carefully avoiding to include seeds deposited on the plant, and stored on silica gel at room temperature until DNA extraction. In large populations, we sampled tissue from 40 adult plants along a transect through the population following the flow direction of the river, at a minimum distance of 2 m between subsequent plants. In small populations, tissue from all individuals was collected. In each population, we recorded GPS coordinates and estimated population size either by counting in small populations, or by counting individuals in a part of the area and extrapolating to the approximate total area; the latter estimates were given as intervals. Interval midpoints were used for regression analyses (see below).

No specific permits were required for the described field studies. The species we are working with, *Myricaria germanica*, is not protected in Switzerland, and therefore, no collecting permit was required. Moreover, we did not collect in the Swiss National Park, other protected areas, or on private land; hence, no permits were required for our field sampling.

DNA was extracted using the DNeasy 96 plant kit (Qiagen). PCR, fragment analyses, and genotyping of 20 nuclear microsatellites were performed as described in [55], excluding Mg461 and Mg482 from the set of 22 loci.

### Data analysis

Gene diversity and allelic richness were calculated using FSTAT version 2.93; to map the values, allelic richness was averaged over the 20 nuclear microsatellites. Population-specific inbreeding coefficients *F*_IS_ and pairwise *F*_ST_ values were calculated with Arlequin version 3.5 [56].

We performed analysis of molecular variance in Arlequin. The *F*-statistics in the AMOVA were based on the number of different alleles, and significance of variance components was tested with 1000 permutations [57]. For the hierarchical AMOVA model, populations were grouped according to the rivers they were collected at. We performed Bayesian analysis of population structure using an admixture model and correlated allele frequencies in Structure version 2.3.3 to define panmictic groups of individuals and to visualize the overall genetic structure in the data [58], [59]. For each value of K ∈ [1], [5], we performed ten replicate simulations using 100,000 iterations as burn-in, followed by 1 million iterations to sample the posterior distributions of parameters. Structure Harvester v. 0.6.8 [60] was utilized to calculate ‘ΔK’, the rate of change in the log probability of the data between successive K values. The K value at which ΔK reaches its maximum is the correct number of clusters [61]. For this value of K, we report the results from the run with highest log likelihood.

In order to depict the genetic relationships between sites, we calculated population graphs in R using the package ‘gstudio’ [62], function ‘population.graph’. Based on graph theory, population graphs can be used to analyze how genetic variability is distributed across space by creating a network of the connections between sites. Nodes are created with size varying based on genetic variability within sites, and a network of connections is identified according to the genetic covariance between sites [63]. Population graphs allow evaluating hypotheses on gene flow between sites by an examination of graph topology. Groups of sites with restricted gene flow can be identified in population graphs from the specific connectivity among edges between groups of sites (e.g. disconnected subnetworks or groups of sites with few connections to other groups of sites). Here, we used the population graph approach to test the hypothesis that groups of sites representing the four main catchments exhibited restricted gene flow. If this were true, we would expect to see four subnetworks representing catchments, each with no or few connections to other subnetworks.

To estimate contemporary migration patterns, we used an assignment method allowing to detect first-generation migrants implemented in the software GeneClass2 version 2.0 [15]. The test identified individuals that had a high probability of originating from another site (e.g. due to gene flow through seed dispersal). Since we had not sampled all potential source populations in the Inn and Rhine catchments, we assessed statistical significance based on the test statistic L_home_, the likelihood of drawing an individual's genotype from the site where it was sampled, given the observed allele frequencies of all sites [14]. The test statistic was computed using Bayesian algorithm [64]. Assignment probabilities were calculated with Monte-Carlo resampling with 1000 permutations according to [14] using a threshold probability of 0.001.

To test specific models about the directionality of gene flow and to obtain Bayesian estimates of effective population sizes and bidirectional rates of migration in *M. germanica*, we used the coalescent-based software Migrate-n version 3.26 [65], [66]. Migrate-n makes the following assumptions: *i*) constant population size through time or random fluctuation around an average size; *ii*) individuals are randomly mating within populations; *iii*) the mutation rate is constant through time and is the same in all parts of the genealogy; *iv*) the immigration rate is constant through time; and *v*) the studied populations exhibit a recent divergence and not an old split, so they exchange material through gene flow [67]. We ran Migrate-n to estimate migration rates among populations (all parameters free to vary) with one long MCMC chain, sampling every 1000^th^ step for a total of 200,000,000 genealogies after a burn-in of 200,000 steps in the chain. A Brownian motion model was used which approximates the stepwise mutation model commonly used for microsatellite data, but converges faster than the standard stepwise mutation model. Populations were randomly resampled to 100 individuals to speed up convergence. Starting parameters for population size θ and migration rates were inferred from *F*_ST_ values; mutation rate modifiers were deduced from the data using Watterson's θ. We performed a series of preliminary runs to explore run conditions and prior distributions for the data from each catchment. Bayesian estimation of migration rate and population sizes were run with one long chain and static heating (temperatures of four Markov chains: 1, 1.5, 3, 10,000,000). The models compared were a full model with all migration rates and population sizes, a stepping-stone model with bidirectional gene flow between neighboring populations, a model where gene flow was directed downstream (but not restricted to neighboring populations), a stepping-stone model where migration occurred only downstream, and a panmictic model. Model selection was performed based on model probabilities and natural log Bayes factors, calculated as LBF = 2(lnmLm_1_-lnmLm_2_), with lnmLm_1_ and lnmLm_2_ being the log marginal likelihoods of model 1 and 2, respectively [68]. We used the marginal likelihoods computed by the Bézier method for all calculations, as these provide precise estimates of marginal likelihoods [68]. LBF>2 suggests preference of model 1 over 2, LBF <−2 suggests preference of model 2 [69]. Model probability was calculated by dividing the marginal likelihood of a given model by the sum of marginal likelihoods of all models [68], [70]. We were only interested in the general direction of migration, i.e. whether upstream and downstream migration rates were substantially different, and thus, we pooled several upstream and downstream populations unless we had sampled few populations in a given catchment. The migration scenarios we could test depended on how many populations a catchment was grouped into. For example, catchments with only two populations allowed only the full, panmictic, and downstream models. As upstream migration (alone) appeared biologically not meaningful, we omitted this type of model. To speed up computation, we ran the parallel version of Migrate-n on a computer cluster using multiple (12–51) cores connected through message passing interface (OpenMPI) [67]. For the purpose of the Migrate-n analyses, the Inn catchment was subdivided into three populations representing upper, middle and lower Inn. The Maggia catchment included two populations. From the Rhone catchment, we omitted a population situated far away on a side river to simplify the models, leaving two populations in the catchment – one large population upstream, and one small population downstream. The Rhine catchment was subdivided into four populations, upper, upper middle, lower middle, and lower Rhine, numbered sequentially from upstream to downstream. Population sizes were transformed to real values by division by the inheritance scalar ×mutation rate, assuming an inheritance scalar of 4 for diploid nuclear data. The mutation rate of nuclear microsatellites is not known for *M. germanica*. We assumed a rate of 10^−3^ [71]; mutation rates for nuclear microsatellite loci range typically from 10^−2^ to 10^−5^ [72]. Migration rates were transformed by multiplication with the transformed population size of the receiving population.

To identify the factors explaining genetic diversity in *M. germanica*, we calculated linear regression models of allelic richness and *F*_IS_ (response variables), affiliation of population to clusters resulting from Bayesian analysis of population structure with software Structure, log10-transformed population size, and elevation in R using the function ‘lm’ [73]. Nine models were compared according to Akaike's information criterion, AIC [74].

#### Isolation by distance

Pairwise estimates of genetic distance are not statistically independent; thus, in this case, significance testing of genetic vs. geographic distance through linear regression is not reliable [18]. Hence, to test the significance of the relationship between genetic and geographic distance, we performed a Mantel test. The Mantel test [75] was performed in R using the function ‘mantel’ implemented in the ‘vegan’ package [76], using pairwise standardized *F*_ST_ values (*F*_ST_/(1−*F*_ST_) and Euclidean geographic distance and Kendall's rank correlation with 999 permutations.

If the genetic data follow a stepping stone model, gene flow should decrease with distance. More specifically, the log_10_ of the gene flow estimate 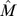 should show a linear decrease with log_10_-transformed geographic distance [19]. Simulation results indicate that in the case of gene flow in one dimension, the slope of the relationship should approximate −1.0, whereas in the two-dimensional case, the slope should approximate −0.3 [19].
