# Correction: Gene Flow within and between Catchments in the Threatened Riparian Plant *Myricaria germanica*

**DOI:** 10.1371/journal.pone.0103669

**Published:** 2014-07-21

**Authors:** The PLOS ONE Staff

The images for Figure 2 and Figure 3 were inadvertently swapped. Please view the correct images and legends for Figure 2 and Figure 3.

**Figure 2:**
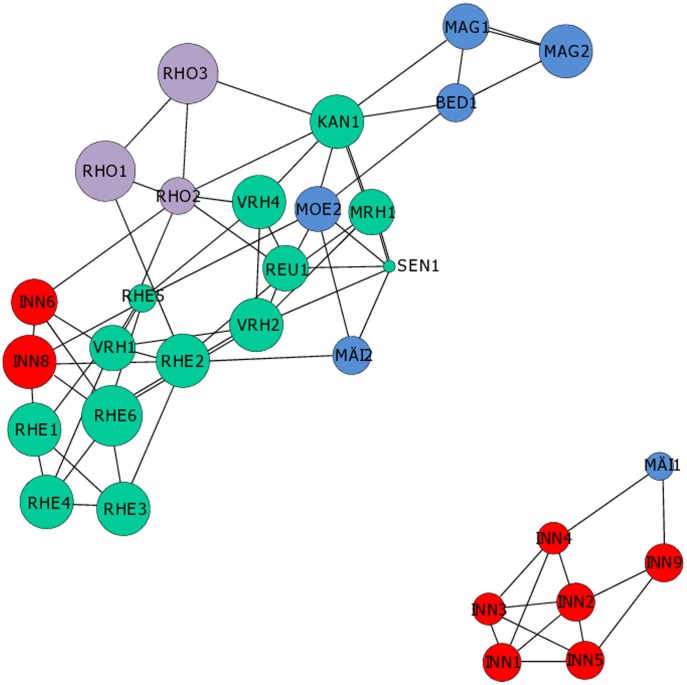
Population graphs showing the genetic relationships between *Myricaria germanica* sites, with catchments shown in different colors (Inn: blue; Rhine, orange; Rhone, red; Ticino, yellow); the size of circles is proportional to effective population size.

**Figure 3:**
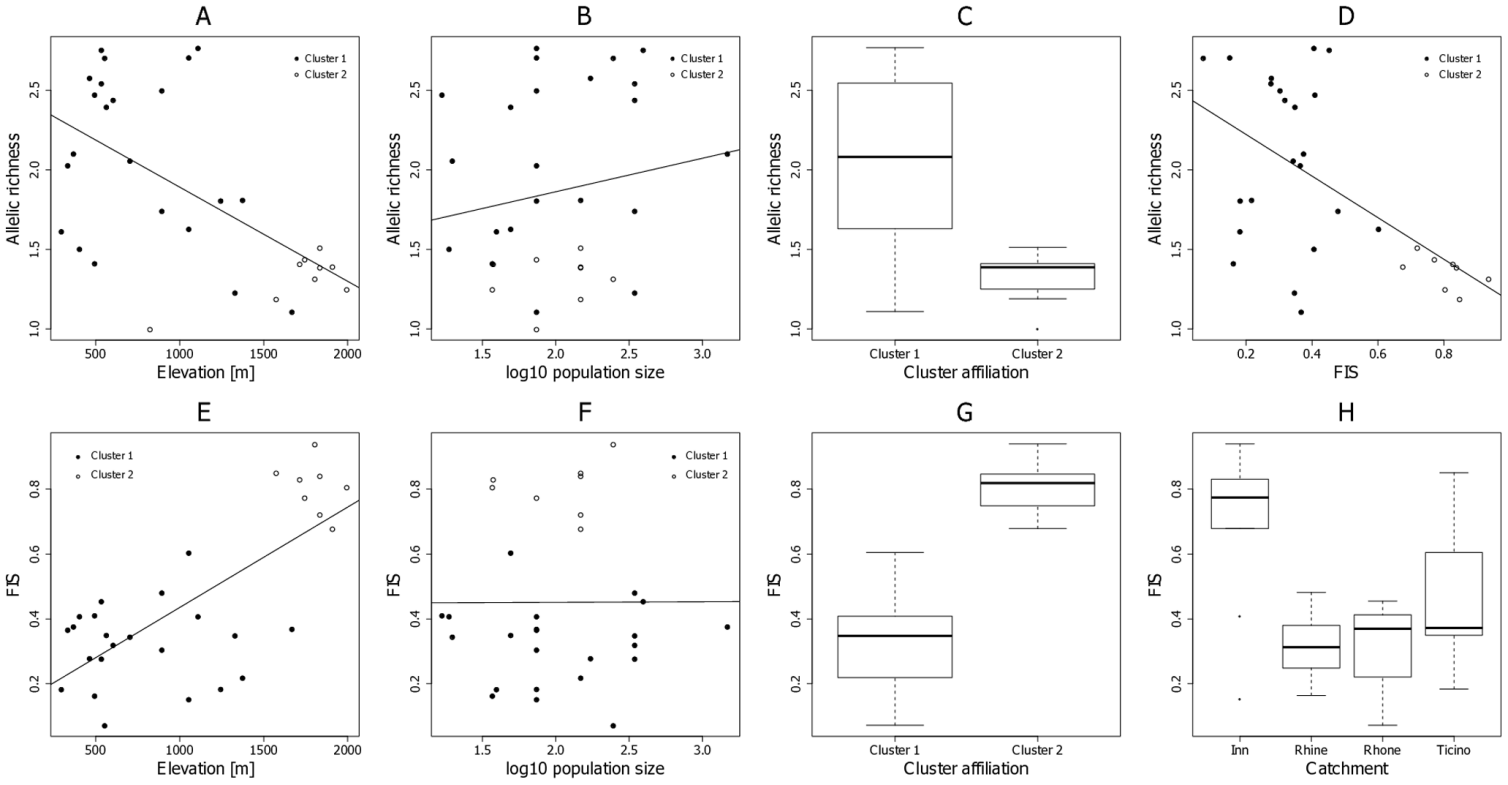
Allelic richness and population-specific inbreeding coefficients (F_IS_) of 20 nuclear SSR in *Myricaria germanica*. A and E. Relationship with elevation. B and F. Relationship with log10-transformed census population size. D. Relationship between allelic richness and F_IS_. C and G. Boxplots of allelic richness, grouped by affiliation to genetic clusters (see Fig. 1B). H. Boxplots of FIS, grouped by affiliation to catchment. The values plotted for census population size are midpoints of the estimated intervals of population size. Lines represent linear regressions. Cluster 1: Rhine, Rhone, Ticino. Cluster 2: Inn.

In addition, some labels were removed from Figure 3 during the production process. The publisher apologizes for this error. Please see the corrected figure above.
